# Calycosin Alleviates Doxorubicin-Induced Cardiotoxicity and Pyroptosis by Inhibiting NLRP3 Inflammasome Activation

**DOI:** 10.1155/2022/1733834

**Published:** 2022-01-05

**Authors:** Lei Zhang, Cundong Fan, Hua-Chen Jiao, Qian Zhang, Yue-Hua Jiang, Jie Cui, Yang Liu, Yong-Hao Jiang, Juan Zhang, Meng-Qi Yang, Yan Li, Yi-Tao Xue

**Affiliations:** ^1^First Clinical Medical College, Shandong University of Traditional Chinese Medicine, Jinan, Shandong 250000, China; ^2^Department of Neurology, Key Lab of Cerebral Microcirculation in Universities of Shandong, Shandong First Medical University & Shandong Academy of Medical Sciences, Taian, Shandong 271000, China; ^3^Cardiovascular Department, Affiliated Hospital of Shandong University of Traditional Chinese Medicine, Jinan, Shandong 250000, China; ^4^Central Laboratory, Affiliated Hospital of Shandong University of Traditional Chinese Medicine, Jinan, Shandong 250000, China; ^5^ICU, Affiliated Hospital of Shandong University of Traditional Chinese Medicine, Jinan, Shandong 250000, China

## Abstract

Calycosin (CAL) is the main active component present in *Astragalus* and reportedly possesses diverse pharmacological properties. However, the cardioprotective effect and underlying mechanism of CAL against doxorubicin- (DOX-) induced cardiotoxicity need to be comprehensively examined. Herein, we aimed to investigate whether the cardioprotective effects of CAL are related to its antipyroptotic effect. A cardiatoxicity model was established by stimulating H9c2 cells and C57BL/6J mice using DOX. *In vitro*, CAL increased H9c2 cell viability and decreased DOX-induced pyroptosis via NLRP3, caspase-1, and gasdermin D signaling pathways in a dose-dependent manner. *In vivo*, CAL-DOX cotreatment effectively suppressed DOX-induced cytotoxicity as well as inflammatory and cardiomyocyte pyroptosis via the same molecular mechanism. Next, we used nigericin (Nig) and NLRP3 forced overexpression to determine whether CAL imparts antipyroptotic effects by inhibiting the NLRP3 inflammasome *in vitro.* Furthermore, CAL suppressed DOX-induced mitochondrial oxidative stress injury in H9c2 cells by decreasing the generation of reactive oxygen species and increasing mitochondrial membrane potential and adenosine triphosphate. Likewise, CAL attenuated the DOX-induced increase in malondialdehyde content and decreased superoxide dismutase and glutathione peroxidase activities in H9c2 cells. *In vivo*, CAL afforded a protective effect against DOX-induced cardiac injury by improving myocardial function, inhibiting brain natriuretic peptide, and improving the changes of the histological morphology of DOX-treated mice. Collectively, our findings confirmed that CAL alleviates DOX-induced cardiotoxicity and pyroptosis by inhibiting NLRP3 inflammasome activation *in viv*o and *in vitro.*

## 1. Introduction

Doxorubicin (DOX) has a wide therapeutic range with demonstrated efficacy against various cancers; however, its dose-dependent cardiotoxicity greatly limits its clinical application. DOX cardiotoxicity can eventually lead to myocardial injury and cardiac failure [1]. To date, the potential mechanism of DOX-mediated cardiotoxicity has mainly focused on oxidative stress, mitochondrial dysfunction, and altered Ca^2+^ homeostasis [2]. Mitochondrial dysfunction increases the generation of intracellular reactive oxygen species (ROS), and oxidative stress remains well-elucidated [3]. In particular, it has been shown that cardiomyocyte death can affect the occurrence and development of DOX-induced cardiotoxicity, including apoptosis, autophagy, necroptosis, ferroptosis, and pyroptosis; however, the precise mechanism needs to be comprehensively examined [4–6]. Although several drugs have been shown to prevent adverse effects of DOX-induced cardiotoxicity [7–9], dexrazoxane is the only U.S. Food and Drug Administration- (FDA-) approved drug for treating DOX-induced cardiotoxicity [10]. Therefore, more effective therapeutic agents and strategies are needed to prevent and manage this challenge.

**Figure 1:**
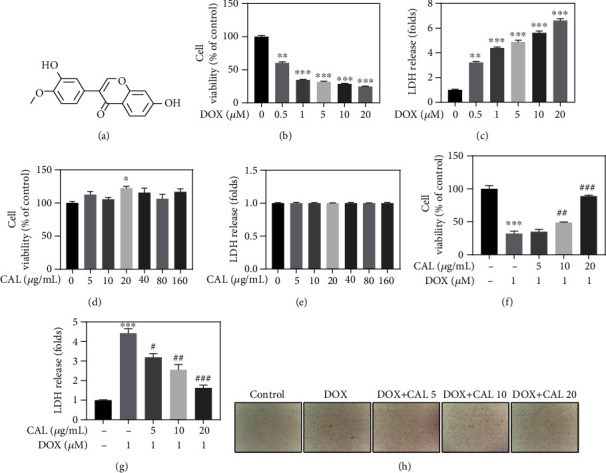
CAL attenuated DOX-induced H9c2 cell injury. (a) Chemical structure of CAL. (b) Cytotoxicity of DOX towards H9c2 cells. Cells were treated with 0-20 *μ*M DOX for 24 h, and cell viability was detected by CCK-8 assay. (c) DOX increased LDH level. (d) Cells were treated with 0-160 *μ*M CAL for 24 h, and cell viability was detected by CCK-8 assay. (e) LDH level was measured in CAL-treated H9c2 cells. (f) CAL improved cell viability in DOX-treated H9c2 cells. (g) CAL decreased LDH release in DOX-treated H9c2 cells. (h) Morphological observation of H9c2 cells. All data were represented as mean ± SEM, *n* = 6. ^∗^*P* < 0.05, ^∗∗^*P* < 0.01, and ^∗∗∗^*P* < 0.001 compared with the control group, ^#^*P* < 0.05, ^##^*P* < 0.01, and ^###^*P* < 0.001 compared with the DOX group.

In traditional Chinese medicine, numerous active components can interact with multiple targets and exhibit minimal side effects, along with significant protective effects against DOX-induced cardiotoxicity [8, 11, 12]. For example, calycosin (C16H12O5, CAL; Figure 1(a)), the main active component present in *Astragalus*, is known to possess anti-inflammatory, antioxidative, antiapoptotic, anticancer, and cardioprotective effects [13, 14]. One study has reported that CAL can ameliorate DOX-induced cardiotoxicity by regulating the Sirt1-NLRP3 pathway to exert a cardioprotective effect, while also reducing cell apoptosis and inhibiting oxidative stress *in vivo* and *in vitro* [13]. In addition, CAL reportedly mediates a protective effect against DOX-induced cardiotoxicity via Atg7-related autophagy [15].

Pyroptosis is a cell death process induced by natural immunity, demonstrating a proinflammatory effect [16]. Pyroptosis can be triggered by pyroptotic caspases, such as caspase-1, -4, -5, and -11, and is closely associated with inflammasome activation. Caspase-1 is activated by innate immune sensors and receptors, such as NLR family pyrin domain 3 (NLRP3); activation of NLRP3 promotes the conversion of interleukin- (IL-) 1*β*, IL-18, and gasdermin D (GSDMD) into their biologically active forms [17]. The NLRP3 inflammasome not only plays an important role in affording protection against bacterial, fungal, and viral infections but has also been linked to cardiovascular diseases such as atherosclerosis, hypertension, coronary heart diseases, heart failure, and myocardial ischemia-reperfusion injury [18]. Thus, the NLRP3 inflammasome is a complex that is associated with cardiovascular disease. Accordingly, the inhibition or antagonism of NLRP3 could prevent cardiovascular diseases. DOX can reportedly induce cardiomyocyte pyroptosis [6, 19]. In addition, DOX treatment has been shown to induce cardiomyocyte pyroptosis via NLRP3/caspase-1 in another study [20]. Given these findings, we focused on developing strategies targeting NLRP3-mediated pyroptosis to improve DOX cardiotoxicity.

Recent studies have revealed that CAL can mitigate DOX-induced cardiotoxicity by inhibiting apoptosis and activating Atg7-related autophagy. However, whether CAL ameliorates DOX-induced cardiotoxicity by inhibiting pyroptosis needs to be clarified. Hence, in the present study, we aimed to explore the effect of CAL on DOX-induced cardiotoxicity and elucidate the underlying molecular mechanisms, focusing on the effect of CAL on NLRP3-mediated pyroptosis in DOX-treated H9c2 cells and C57BL/6J mouse models.

## 2. Materials and Methods

### 2.1. Materials

DOX (#MB1087) was purchased from Meilun Biotechnology (Dalian, China). CAL was obtained from DASF Biotechnology (Nanjing, China). Nigericin (#NSC 292567) was purchased from Selleck (Houston, TX, USA). pcDNA-NLRP3 and empty pcDNA were synthesized by Tsingke Biotechnology Co., Ltd. (Beijing, China).

NLRP3 (#19771-1-AP) was purchased from Proteintech. ASC (#A1170), caspase-1 (#A0964), IL-1*β* (#A1112), IL-18 (#A16737), and GAPDH (#AC001) were purchased from Abclonal. GSDMD (#ab209845) was purchased from Abcam.

### 2.2. Cell Culture and Drug Treatment

H9c2 cells were purchased from the Cell Bank of the Chinese Academy of Sciences (Shanghai, China). H9c2 cells were maintained in modified RPMI medium (#SH30809.01, HyClone Co. Ltd, USA), supplemented with 10% certified fetal bovine serum (#04-001-1ACS, BI Co. Ltd, Israel), 1% penicillin, and streptomycin, at 37°C in a 5% CO2 incubator. H9c2 cells were stimulated with CAL (98% purity) for 1 h, followed by DOX for 24 h. In some experiments, cells were pretreated with the pcDNA-NLRP3 plasmid constructs for 20 h or with nigericin for 1 h prior to CAL treatment.

#### 2.2.1. Cell Viability Assay

Cell Counting Kit-8 (CCK-8, #A311-02-A, Vazyme Bio-technology Co., Ltd, Nanjing, China) was used to measure cell viability in a simple and efficient manner, in accordance with the manufacturer's instructions. Briefly, H9c2 cells were treated with CAL (5-160 *μ*g/mL) and/or DOX (0.5-20 *μ*M) and/or nigericin (0.5-20 *μ*M) for 24 h. Next, H9c2 cells were treated with CCK-8 solution for another 1 h. Absorbance was measured at 450 nm using a microplate reader (Imark-22353, BIO-RAD, USA).

#### 2.2.2. Adenosine Triphosphate (ATP) Measurements

Cells were divided into five groups: (1) control group, (2) DOX group, (3) DOX + CAL (5 *μ*g/mL), (4) DOX + CAL (10 *μ*g/mL), and (5) DOX + CAL (20 *μ*g/mL). H9c2 cells were collected, lysed, and centrifuged. Proteins were subsequently extracted, and their concentrations were determined using a bicinchoninic acid protein kit (BCA, #PC0020, Solarbio Biotechnology Co., Ltd, Beijing, China). The sample or standard protein supernatant (20 *μ*L) was then mixed with an ATP assay kit (#S0026, Beyotime, Shanghai), and fluorescence was measured using a microplate reader (Spark, TECAN, Austria).

#### 2.2.3. Assay for Intracellular ROS

After cells were grouped as described above, the ROS assay kit (#S0033S, Beyotime, Shanghai) was used to assess intracellular ROS levels using the 2′,7′-dichlorofluorescein diacetate (DCFH-DA) method. Briefly, H9c2 cells were stained with DCFH-DA for 30 min in the dark. Next, cells were examined using a fluorescence microscope. ImageJ software (National Institutes of Health, Bethesda, MD) was used to examine the fluorescence density of ROS. Intracellular ROS was detected by flow cytometry (#C6 Plus, BD, USA).

#### 2.2.4. JC-1 Mitoscreen Assay

Mitochondrial injury was detected using the JC-1 assay kit (#C2006, Beyotime, Shanghai) in accordance with the manufacturer's instructions. The cells were incubated with the JC solution and analyzed using a fluorescence microscope. Mitochondrial membrane potential (MMP) was determined using flow cytometry (#C6 Plus, BD).

#### 2.2.5. Analysis of Oxidative Stress

After cells were grouped according to the above treatment, the malondialdehyde (MDA) content was measured using the Lipid Peroxidation MDA assay kit (#S0131S). The glutathione peroxidase (GSH-Px) content was detected using a glutathione peroxidase assay kit (#S0058), while superoxide dismutase assay kit (#S0101S) was used to evaluate the superoxide dismutase (SOD) activity. All the kits were purchased from Beyotime (Shanghai, China).

#### 2.2.6. Immunofluorescence

Fix & Perm (MultiSciences, Hangzhou, China) was added after cell centrifugation. After incubation with 5% bovine serum albumin, H9c2 cells were incubated with primary antibodies (NLRP3 and caspase-1), and secondary antibodies were incubated overnight at 4°C. Then, H9c2 cells were stained with DAPI (#S0033S, Beyotime) for 10 min. An antifluorescence quenching sealing tablet (#S2100, Solarbio, China) was used to seal the film, and a fluorescence microscope (Nikon NI-V) was used to obtain representative images.

### 2.3. Animals and Treatments

Herein, we used 45 C57BL/6J male mice, 8-week-old and weighing 18–22 g, purchased from the Vital River Laboratory Animal Technology (Zhejiang, China). All experimental animals were reared under standard laboratory conditions of 55 ± 5% relative humidity, 23 ± 2°C, and 12 h light. The experimental protocol was approved by the ethics committee of the Affiliated Hospital of Shandong University of Traditional Chinese Medicine (Permission number: 2021-10) and conducted in accordance with the National Institute of Health's Guide for the Care and Use of Laboratory Animals (#8023, United States).

To examine the effect of CAL on DOX-induced cardiotoxicity, mice were divided into three experimental groups (*n* = 15), as follows.

Control group: mice were intraperitoneally injected with normal saline at the same volume and frequency as DOX and CAL; DOX group: mice were intraperitoneally injected with 5 mg/kg DOX weekly for 4 weeks and injected with normal saline at the same volume and frequency as CAL; CAL + DOX group: mice were injected intraperitoneally with 5 mg/kg DOX weekly for 4 weeks, and intraperitoneally injected with 50 mg/kg body weight of CAL every other day. Echocardiography was performed on day 29 of the experiment; the mice were deeply anesthetized with pentobarbital sodium and immediately sacrificed to harvest the blood and heart.

#### 2.3.1. Echocardiography

Echocardiography was performed with a portable color Doppler ultrasound scanner Mindray M5 echocardiographic transducer (#2P2s) of 2–4 MHz to evaluate the effect of CAL on cardiac structure and function. The mice were deeply anesthetized, with chest hairs were shaved, and placed on a heated platform to perform the ultrasound examination. The operators were blinded to the grouping and treatment of the experimental mice.

#### 2.3.2. Heart Tissue Histopathology and Immunohistochemical Analysis

As previously detailed [21], hematoxylin and eosin (H&E) staining and Masson's trichrome staining were performed using paraformaldehyde-fixed and paraffin-embedded cardiac tissue samples to confirm cardiomyocyte morphology, inflammatory cell infiltration, and myocardial fibrosis degree.

TUNEL and immunohistochemical (IHC) staining were performed following the standard protocol. TUNEL staining was used to detect the number of TUNEL-positive cells; immunohistochemical staining was performed to determine the NLRP3 expression. TUNEL-positive cells showed brown staining granules in the nucleus, and NLRP3 protein expression exhibited brown, yellow granules in the cytoplasm.

Representative images were captured using a Nikon Ni-V microscope and evaluated using ImageJ software (version 5.0).

### 2.4. Lactate Dehydrogenase (LDH) Release Assay

H9c2 cells were stimulated with DOX (0.5-20 *μ*M) and/or CAL (5-160 *μ*g/mL) and/or nigericin (0.5-20 *μ*M), and mice were treated with CAL or/and DOX. According to the manufacturer's instructions, the LDH cytotoxicity assay kit (#C0016, Beyotime, Shanghai) was used to measure LDH levels in serum and cell supernatant. For detecting LDH levels in the cell supernatant, the H9c2 cell supernatant was transferred to a 96-well plate, and the detection solution (60 *μ*L) was added to each well. Serum (10 *μ*L) was added to the sample well for testing, and 40 *μ*L of sample dilution was then added. After incubating at room temperature in the dark for 30 min, absorbance was measured and detected.

### 2.5. ELISA Analysis

Serum and cell supernatants were collected after different treatments of cells and mice. As described previously [21], ELISA kits were used to detect serum levels of brain natriuretic peptide (BNP), monocyte chemotactic protein 1 (MCP-1), and C-reactive protein (CRP), as well as concentrations of IL-1*β* and IL-18 in the cell supernatant, according to the manufacturer's instructions.

### 2.6. Western Blotting Analysis

Protein lysates were harvested from cultured H9c2 cells and cardiac tissue samples. The total protein concentrations in H9c2 cells and mouse heart samples were determined using the BCA Kit. Western blotting was performed according to standard methods. Membranes were incubated with primary antibodies against NLRP3, ASC, caspase-1, IL-1*β*, IL-18, GSDMD, and GAPDH. Next, the cells were incubated with a secondary antibody solution. ECL substrate chemiluminescence solution (#E412-02, Vazyme Bio-technology Co., Ltd., Nanjing, China) was uniformly spread onto membranes. The gray value of each band was quantified using the ImageJ software.

### 2.7. Statistical Analysis

Data values are expressed as means ± standard error of the mean (SEM). Student's *t*-test and one-way ANOVA followed by Dunnett's post hoc test were employed to analyze statistical significance. Data were analyzed using GraphPad Prism version 5.0 (GraphPad Software, Inc., San Diego, CA, USA), and statistical significance was set at *P* < 0.05.

## 3. Results

### 3.1. CAL Attenuated DOX-Induced H9c2 Cell Injury

To determine whether CAL attenuates DOX-induced cell injury, the CCK-8 assay and LDH release were first examined to evaluate its protective effect *in vitro*. First, treatment of H9c2 cells with different concentrations (0–20 *μ*M) of DOX for 24 h resulted in dose-dependent inhibition of H9c2 cell viability and increased LDH release (Figures 1(b) and 1(c)). Given these results and previous clinical studies [22], 1 *μ*M DOX was used in the following *in vitro* experiments to construct a stable cardiotoxicity model. Subsequently, CAL treatment exhibited no cytotoxicity towards H9c2 cells, while effectively alleviating DOX-induced H9c2 cell damage and death in a dose-dependent manner, especially at 20 *μ*g/mL, as evidenced by cell viability, LDH release, cell morphology, and cell number (Figures 1(d)–1(h)). Accordingly, these results indicated that CAL could attenuate DOX-induced H9c2 cell injury.

### 3.2. CAL Suppressed DOX-Induced Mitochondrial Injury and Oxidative Stress in H9c2 Cells

Mitochondria are a well-known source of cellular energy, and recent studies have found that mitochondria play an indispensable role in cell survival. As mitochondria are the most severely damaged intracellular organelles following DOX exposure, we investigated whether CAL could reduce DOX-induced mitochondrial oxidative stress injury.

**Figure 2:**
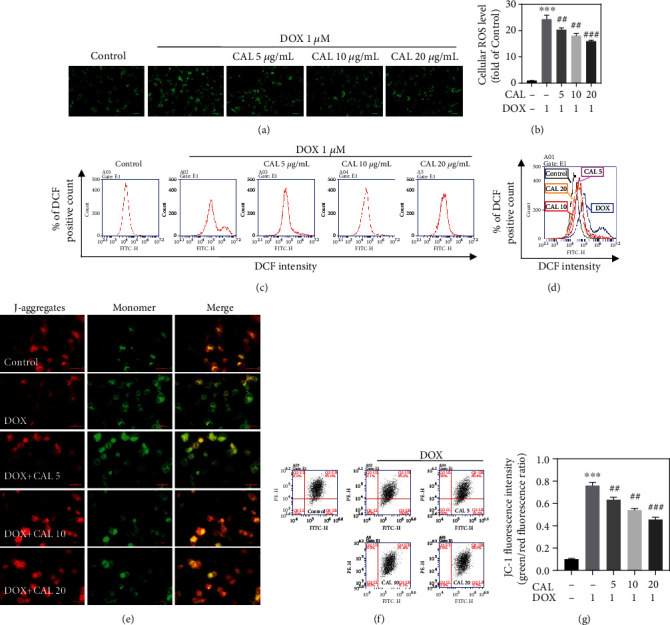
Effects of CAL on DOX-induced mitochondrial oxidative stress injury in H9c2 cells. (a) ROS generation in H9c2 cells was assayed by 2′,7′-dichlorofluorescein–diacetate (DCFH2–DA) staining. Images were obtained using a fluorescence microscope (magnification, ×100; scale bar, 100 *μ*m) with DCF-positive cells were green. (b) Quantitative analysis of DCF fluorescence intensity (*n* = 3). (c) Fluorescence intensity measured by flow cytometry. (d) Overlay flow cytometry histograms of cultures. (e) Representative images of mitochondrial membrane potential in H9c2 cells (magnification, ×100; scale bar, 100 *μ*m). (f) Mitochondrial membrane potential measured by flow cytometry. (g) Quantitative analysis of JC-1 fluorescence intensity (*n* = 3). All data were represented as mean ± SEM. ^∗^*P* < 0.05, ^∗∗^*P* < 0.01, ^∗∗∗^*P* < 0.001 compared with the control group, ^#^*P* < 0.05, ^##^*P* < 0.01, ^###^*P* < 0.001 compared with the DOX group.

ROS accumulation is the primary biological event during DOX-induced myocardial injury. Hence, ROS accumulation was first examined by DCFH-DA staining and flow cytometry (Figures 2(a) and 2(c)). We observed that DOX increased intracellular ROS levels, and CAL inhibited this effect in a dose-dependent manner (Figures 2(b) and 2(d)). In addition, to estimate the integrity of the mitochondrial membrane, MMP (ΔΨ*m*) was evaluated using JC-1 staining and flow cytometry. CAL significantly reduced DOX-induced MMP reduction in H9c2 cells in a dose-dependent manner (Figures 2(e)–2(g)), indicating that CAL pretreatment prevented mitochondrial membrane damage attributed to DOX treatment. Loss of MMP results in depletion of ATP (cell energy), ultimately resulting in cell death [23]. As shown in Supplementary Figure 1(a), DOX treatment reduced the ATP content in H9c2 cells. However, CAL cotreatment increased ATP content in a dose-dependent manner.

The levels of MDA, SOD, and GSH-Px were measured to determine the effect of CAL on DOX-mediated oxidative stress in H9c2 cells. In the present study, DOX stimulation significantly enhanced the MDA content and suppressed SOD and GSH-Px activities *in vitro*. Interestingly, CAL treatment restored the DOX-induced increase in MDA content and decrease in SOD and GSH-Px activities *in vitro* (Supplementary Figure 1(b)–1(d)). Therefore, these findings suggested that CAL reduced DOX-induced mitochondrial injury and oxidative stress *in vitro*.

### 3.3. CAL Prevented NLRP3 Inflammasome-Mediated Pyroptosis in DOX-Treated H9c2 Cells

**Figure 3:**
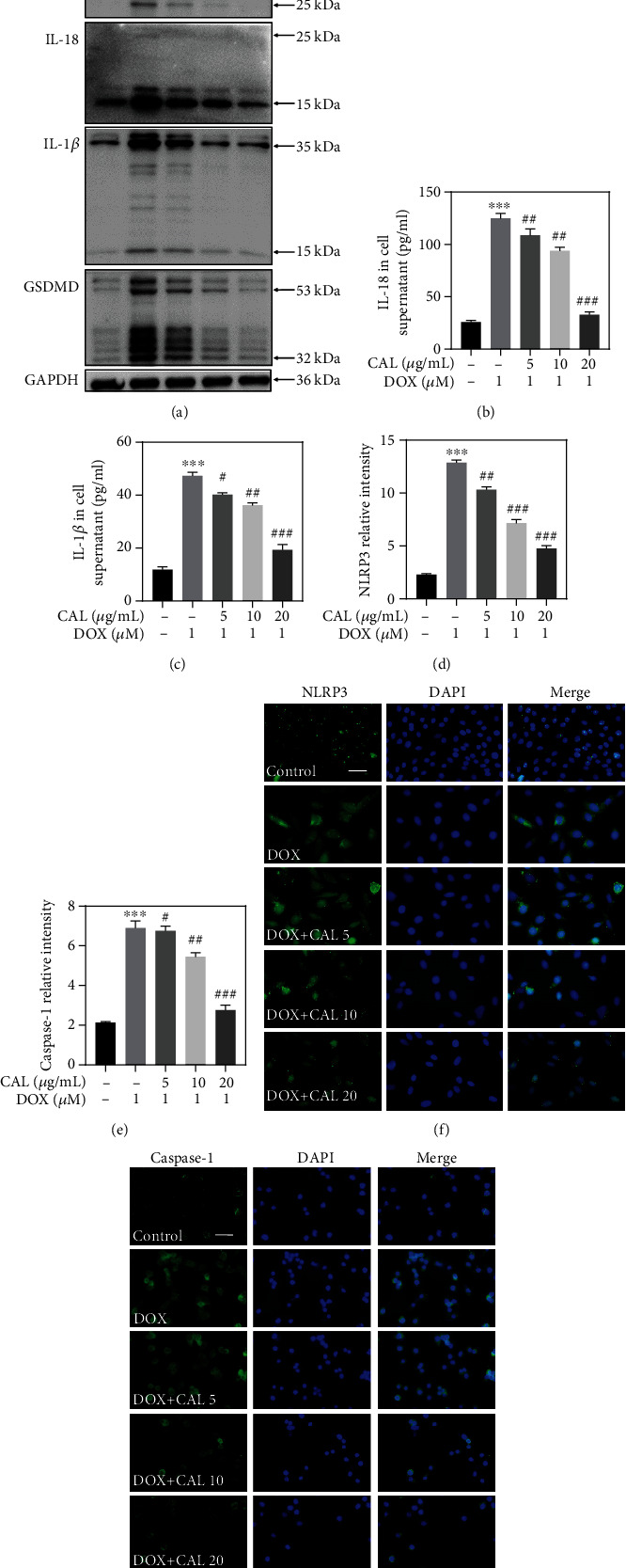
CAL prevented DOX-induced cardiomyocyte pyroptosis in H9c2 cells. (a) Expressions of NLRP3, ASC, caspase-1, IL-1*β*, IL-18, and GSDMD in H9c2 cells. Protein expression was examined by western blotting. (b) Content of IL-18 in H9c2 cell supernatant (*n* = 6). (c) Content of IL-1*β* in H9c2 cell supernatant (*n* = 6). (d) and (e) Quantitative analysis of NLRP3 and caspase-1 expression (*n* = 3). (f) and (g) Representative immunofluorescent staining of NLRP3 and caspase-1. All data were represented as mean ± SEM. ^∗∗∗^*P* < 0.001 vs. control group, ^#^*P* < 0.05, ^##^*P* < 0.01, ^###^*P* < 0.001 vs. DOX group.

NLRP3-triggered inflammatory response and pyroptosis play a key role in DOX-induced cardiotoxicity [20]. Hence, NLRP3-triggered pyroptosis and inflammation were examined *in vitro* to further explore the potential molecular mechanism underlying the cardioprotective effect of CAL. First, western blotting was used to assess the expression of related proteins in the pyroptosis pathway. As shown in Figure 3(a), DOX treatment significantly induced the activation of NLRP3 and ASC, cleavage of caspase-1, GSDMD, IL-1*β*, and IL-18 proteins *in vitro*. As expected, cotreatment with CAL and DOX inhibited protein levels in a dose-dependent manner, especially at 20 *μ*g/mL. The quantitative analysis further confirmed that CAL inhibited the NLRP3-mediated pyroptosis pathway (Supplementary Figure 1(e)–1(j)). Furthermore, previous studies have shown that pyroptosis can promote inflammation [24], and we investigated whether CAL affected inflammation *in vitro* following DOX treatment. As depicted in Figures 3(b) and 3(c), DOX treatment of H9c2 cells induced the release of IL-18 and IL-1*β*; however, CAL cotreatment reduced IL-18 and IL-1*β* release. Lastly, to further determine the effect of CAL on NLRP3-mediated pyroptosis, immunofluorescence assays of NLRP3 and caspase-1 were performed. The staining results revealed that NLRP3 and caspase-1 expression increased following DOX exposure. However, immunofluorescence of cells treated with CAL and DOX showed a significant dose-dependent decrease in NLRP3 and caspase-1 expression (Figures 3(f) and 3(g)). Furthermore, quantitative analysis of immunofluorescence confirmed that CAL inhibited the expression of the NLRP3-mediated pyroptosis-associated protein (Figures 3(d) and 3(e)), consistent with western blotting results. Collectively, these findings indicated that CAL could prevent DOX-induced pyroptosis in H9c2 cells.

### 3.4. NLPR3 Reversed the Anticardiotoxicity Effects of CAL In Vitro

To confirm that CAL inhibits DOX-induced cardiotoxicity via the NLRP3 inflammasome, nigericin (a special stimulator of NLRP3) and CAL (20 *μ*g/mL) were employed. The effect of nigericin on the viability of H9c2 cells was determined using the CCK8 assay. The results revealed that nigericin was highly toxic to H9c2 cells. As shown in Supplementary Figure 2(a), different nigericin concentrations (0, 0.5, 1, 5, 10, 20, and 40 *μ*M) inhibited the viability of H9c2 cells in a dose-dependent manner. Subsequently, similar to the results of the previous experiment, CAL (20 *μ*g/mL) effectively alleviated DOX-induced H9c2 cell damage and death, as shown by the improved cell viability, decreased LDH release, improved cell morphology, and increased cell number. However, nigericin reversed these CAL-induced changes (Supplementary Figure 2(b)–2(d)). Overall, these results indicated that CAL exerts cardioprotective effects by inhibiting the NLRP3 inflammasome.

### 3.5. NLPR3 Reversed the Antipyroptotic Effects of CAL In Vitro

**Figure 4:**
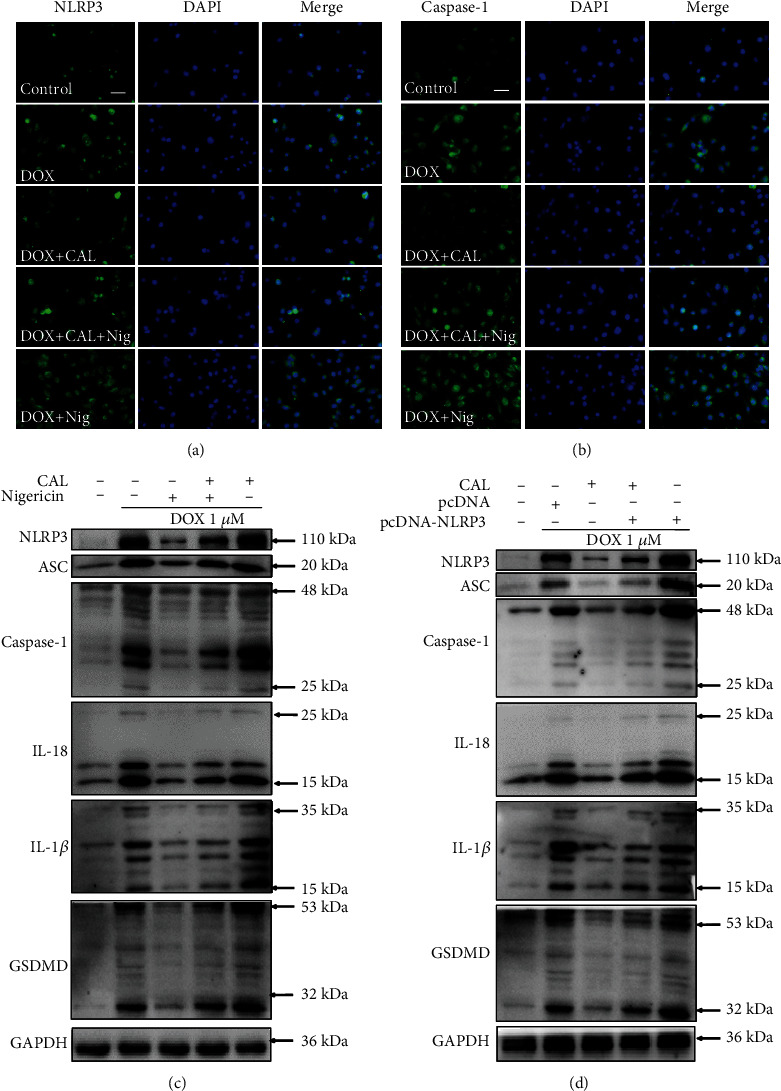
Activation of NLRP3 with nigericin and overexpression of NLRP3 reversed the antipyroptotic effects of CAL *in vitro*. (a) and (b) Representative immunofluorescent staining of NLRP3 and caspase-1. (c) NLRP3 activation by nigericin abolished CAL anti-pyroptotic effects *in vitro*. Representative Western blot images of NLRP3, ASC, Caspase-1, IL-1*β*, IL-18, and GSDMD in H9c2 cells. (d) Forced overexpression of NLRP3 reversed CAL antipyroptotic effects *in vitro*. Protein expressions of NLRP3, ASC, caspase-1, IL-1*β*, IL-18, and GSDMD in H9c2 cells were examined by Western blotting.

To verify the hypothesis that CAL affects pyroptosis by regulating the NLRP3 inflammasome, nigericin and NLRP3 overexpression were combined with CAL treatment. As depicted in Supplementary Figure 2(e)–2(f), cotreatment with nigericin significantly reversed the CAL-mediated inhibition of IL-18 and IL-1*β* release induced by DOX in cell supernatants, indicating that NLRP3 activation promotes inflammation. In addition, as shown in Figure 4(c), similar to the previous experiment, CAL-DOX cotreatment significantly inhibited the activation of NLRP3 and ASC, cleavage of caspase-1, IL-1*β*, IL-18, and GSDMD proteins; however, nigericin restored these protein levels. Quantitative analysis confirmed this result (Supplementary Figure 3(a)). As observed in the previous experiment, immunofluorescent staining was performed for NLRP3 and caspase-1, which are pyroptotic markers (Figures 4(a) and 4(b)). The immunofluorescence staining results of NLRP3 and caspase-1 were consistent with those of western blotting (Supplementary Figure 2(g), 2(h)). Lastly, to further investigate whether the impact of CAL on pyroptosis occurred in an NLRP3-dependent manner *in vitro*, NLRP3 was forcibly overexpressed. Western blotting revealed that NLRP3 overexpression reversed the inhibitory effect of CAL on the expression of pyroptotic-related proteins, indicating that CAL alleviates pyroptosis by inhibiting the NLRP3 inflammasome (Figure 4(d)); quantitative analysis confirmed this finding (Supplementary Figure 3(b)). In summary, the above results suggested that CAL affords cardioprotection and alleviates pyroptosis by inhibiting the NLRP3 inflammasome.

### 3.6. CAL Alleviated DOX-Induced Cardiac Injury in Mice

**Figure 5:**
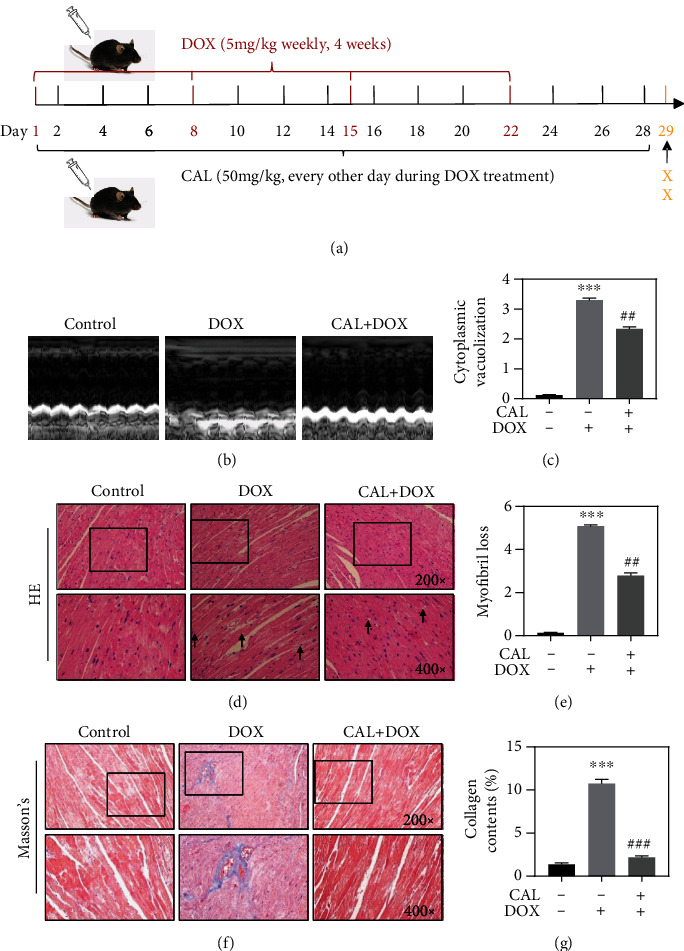
CAL alleviated DOX-induced cardiac dysfunction and myocardial injury in mice. (a) Schematic diagram of the experimental design. (b) Representative images of echocardiography tracing of mice treated with DOX and CAL at 29 days. (c) Vacuolation of cytoplasm by HE staining and quantified (*n* = 3). (d) Representative HE staining of mice heart. The representative degenerative vacuoles were indicated with arrowhead. (e) Myofibril loss by HE staining and quantified (*n* = 3). (f) Representative Masson trichrome staining of mice heart. (g) Collagen contents (*n* = 3). Data were depicted as mean ± SEM. ^∗∗^*P* < 0.01, ^∗∗∗^*P* < 0.001 compared with the control group, ^#^*P* < 0.05, ^##^*P* < 0.01, ^###^*P* < 0.001 compared with the DOX group.

To determine the effect of CAL on cardiac damage caused by DOX *in vivo*, we treated mice with CAL and examined the cardioprotection after 28 days, as shown in Figure 5(a). We observed that CAL treatment reduced the increase in the heart weight to body weight ratio induced by DOX (Supplementary Figure 4(a)). Furthermore, cardiac function was determined to evaluate cardiac injury (Figure 5(b)). As shown in Supplementary Figure 4(b)–(e), CAL treatment reversed the DOX-induced decline in cardiac function, as evidenced by the elevated left ventricular ejection fraction (LVEF), left ventricular fractional shortening (LVFS), and left ventricular end-systolic diameter (LVESD) and left ventricular end-diastolic diameter (LVEDD). Next, we examined serum BNP, another key biomarker related to cardiac function. As shown in Supplementary Figure 4(f), DOX treatment-induced myocardial injury leads to significantly increased serum BNP levels; however, CAL effectively reduced the BNP level. Meanwhile, serum LDH was significantly upregulated in DOX-treated mice; however, cotreatment with CAL and DOX attenuated LDH release (Supplementary Fig. 4(g)), consistent with BNP results.

Given the importance of inflammation in DOX-mediated myocardial injury, serum CRP and MCP-1 levels were evaluated. We observed that both serum CRP and MCP-1 levels were increased in response to DOX exposure. However, CAL markedly reduced serum CRP and MCP-1 levels in DOX-treated mice (Supplementary Fig. 4(h), (i)). Therefore, these results indicated that CAL alleviates DOX-induced inflammatory responses *in vivo*.

Next, we investigated whether CAL could afford beneficial effects on the cardiac histopathology of mice. Changes in the histological morphology of the heart were confirmed by H&E and Masson's trichrome staining (Figures 5(d) and 5(f)). H&E staining revealed that the myocardial tissue structure was notably disordered after DOX treatment, with inflammatory cell infiltration, increased cytoplasmic vacuoles, and myofibrillary loss. CAL cotreatment effectively ameliorated these changes in histological morphology (Figures 5(c) and 5(e)). In addition, quantitative analysis of Masson's trichrome staining showed that CAL treatment decreased the area of myocardial fibrosis (Figure 5(g)). Collectively, these results strongly indicated that CAL affords a protective effect against DOX-induced cardiac injury *in vivo*.

### 3.7. CAL Inhibited NLRP3 Inflammasome-Mediated Cardiomyocyte Pyroptosis in DOX-Treated Mice

**Figure 6:**
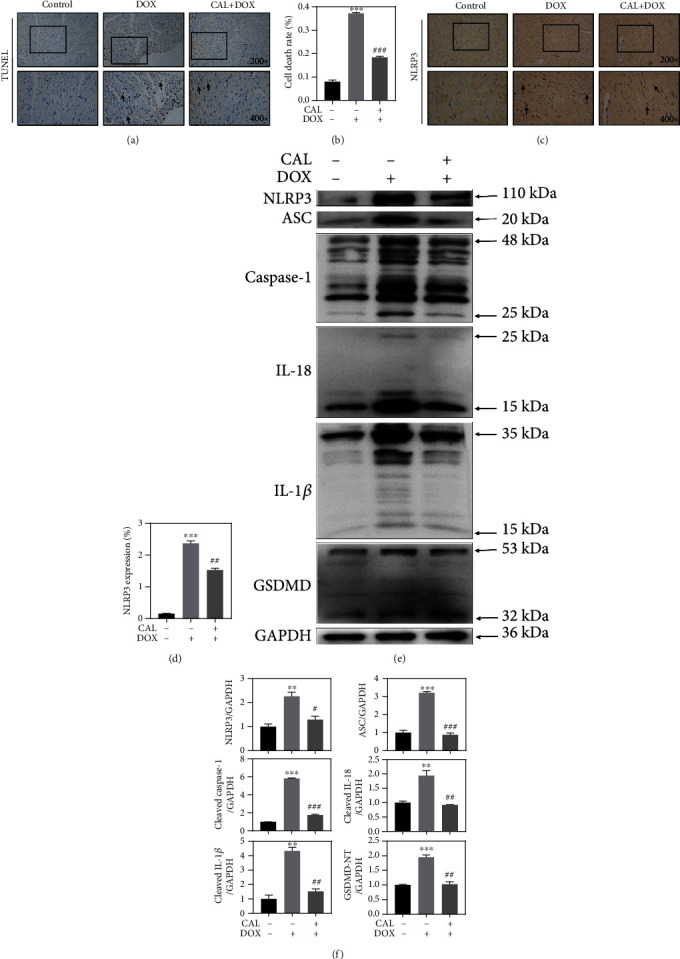
CAL suppressed DOX-induced cardiomyocyte pyroptosis in mice. (a) Representative TUNEL staining of mice heart. The representative TUNEL positive cells were indicated with arrowhead. (b) Statistics analysis was performed to quantify the cardiomyocyte death (*n* = 4). (c) Representative images of NLRP3 immunohistochemistry. The representative NLRP3 positive cells were indicated with arrowhead. (d) Quantitative immunohistochemistry analysis of NLRP3 (*n* = 4). (e) Representative Western blot images of NLRP3, ASC, caspase-1, IL-1*β*, IL-18, and GSDMD expression in mouse myocardium. (f) Quantitative analysis of NLRP3, ASC, caspase-1, IL-1*β*, IL-18, and GSDMD in mouse myocardium (*n* = 3). GAPDH was used as loading control. Data were expressed as mean ± SEM. ^∗^*P* < 0.05 and ^∗∗∗^*P* < 0.001 compared with the control group, ^#^*P* < 0.05, ^##^*P* < 0.01, and ^###^*P* < 0.001 compared with the DOX group.

We have previously confirmed that CAL improved DOX-induced cardiotoxicity by inhibiting pyroptosis *in vitro*. Accordingly, we further examined whether CAL treatment could exert a suppressive effect on DOX-induced cardiomyocyte pyroptosis in C57BL/6J mice. The TUNEL assay confirmed the protective effect of CAL on cardiomyocytes (Figure 6(a)). Quantitative analysis of TUNEL revealed that the number of TUNEL-positive cells was increased after DOX treatment; however, this effect was attenuated by CAL treatment *in vivo* (Figure 6(b)). The expression of NLRP3 was measured by IHC analysis (Figure 6(c)). As expected, quantitative IHC analysis revealed that NLRP3 expression was markedly increased following DOX administration; however, CAL treatment significantly alleviated the expression of NLRP3 (Figure 6(d)). Moreover, western blotting was performed on pyroptosis-mediated protein markers to confirm the suppressive effect of CAL cotreatment on cardiomyocyte pyroptosis. As expected, DOX injection significantly caused activation of NLRP3, ASC, and cleavage of caspase-1, GSDMD, IL-1*β*, and IL-18 in mice. However, CAL cotreatment remarkably attenuated DOX-induced activation and cleavage of these proteins *in vivo* (Figure 6(e)). Quantitative analysis of western blotting results further corroborated these findings (Figure 6(f)). Overall, these data provide evidence that CAL treatment could mediate a suppressive effect on DOX-induced cardiomyocyte pyroptosis *in vivo*.

## 4. Discussion

In the present study, we demonstrated that CAL significantly ameliorates DOX-induced cardiotoxicity via antioxidant, anti-inflammatory, and antipyroptotic effects. Specifically, CAL protected cardiomyocytes from pyroptosis by inhibiting NLRP3 inflammasome activation.

Although DOX has been recognized as one of the most effective and safe anticancer drugs, its clinical use has been limited owing to its high toxicity, especially cardiotoxicity [25]. Currently, several pathogenic mechanisms have been implicated in DOX-induced cardiotoxicity, such as oxidative stress, mitochondrial dysfunction, inflammation, autophagy, apoptosis [26], pyroptosis [27], and ferroptosis [28]; however, the findings remain controversial. As shown in previous studies, DOX caused cardiac dysfunction and cardiotoxicity. Herein, *in vitro* experiments revealed that DOX inhibited H9c2 cell viability and increased LDH release; CAL effectively alleviated DOX-induced H9c2 cell damage and death in a dose-dependent manner, as evidenced by cell viability, LDH release, cell morphology, and cell number. In the *in vivo* experiment, CAL cotreatment partially reversed the damage to cardiac function, reduced BNP levels, alleviated the inflammatory response, and improved histological morphology in DOX-treated mice. Collectively, the DOX-induced damage to cardiomyocytes was substantially attenuated following CAL treatment *in vivo* and *in vitro*. More importantly, the cardioprotective effect of CAL observed in this study could be attributed to the inhibition of ROS production, inflammation, and pyroptosis.

Mitochondrial-derived ROS are related to pathological processes of several heart diseases, including diabetic cardiomyopathy and heart failure [29, 30]. Oxidative stress, such as inflammation, is also closely related to DOX-induced cardiotoxicity [31]. DOX-derived ROS can lead to mitochondrial injury and subsequently cause cardiomyocyte death. Accumulating evidence has demonstrated that one of the main mechanisms underlying DOX-induced cardiotoxicity is oxidative stress, defined as an imbalance between the increase in ROS generation and the decrease in antioxidants [32]. Mitochondria (energy metabolism center) and MMP (ΔΨ*m*) play important roles in cell death. The loss of MMP (ΔΨ*m*) causes an increase in ATP consumption, ultimately resulting in cell death [23]. We further studied mitochondrial function and oxidative stress to comprehensively explore the cardioprotective effects of CAL. In the present study, DOX elevated ROS levels, whereas CAL pretreatment reduced ROS levels. DOX causes a loss of ΔΨ*m*; however, CAL pretreatment enhances ΔΨ*m*. In addition, CAL pretreatment enhanced the ATP content, consistent with the altered ΔΨ*m*. CAL-DOX cotreatment decreased the MDA content and increased SOD and GSH-Px activities in H9c2 cells. However, whether the antioxidant effect of CAL is related to pyroptosis needs to be elucidated.

DOX-induced cardiotoxicity is known to be associated with inflammation [33]. Increasing evidence has shown that DOX can cause the activation of the NLRP3 inflammasome, leading to a series of inflammatory reactions and cardiotoxicity. However, after the appearance of cardiotoxicity, inflammation is aggravated [34]. We observed that DOX treatment exacerbated the levels of inflammatory factors (NLRP3, CRP, MCP-1, IL-1*β*, and IL-18), whose expression affected DOX-induced H9c2 cardiomyocyte toxicity and death. However, CAL treatment relieved the inflammatory response. Based on our findings, we speculate that CAL can protect cardiomyocytes from DOX damage by inhibiting the expression of inflammatory factors, mainly confirmed by decreased NLRP3 inflammation and systemic inflammation.

Preventing myocardial cell death is considered an effective cardioprotective strategy [35]. Pyroptosis is an important natural immune response that is closely associated with proinflammatory effects [16]. In contrast to apoptosis, the classical pyroptotic pathway depends on activated caspase-1 to hydrolyze the membrane protein GSDMD. GSDMD-NT is known to participate in the formation of pore membrane channels, which can destroy the cell membrane integrity. Therefore, pyroptosis is accompanied by cell swelling and the release of intracellular proinflammatory cytokines [17]. Recently, pyroptosis was shown to be involved in the occurrence and development of cardiovascular diseases [36]. However, there is insufficient evidence regarding the effect of pyroptosis on DOX-induced cardiotoxicity. To confirm our hypothesis, we treated H9c2 cells with DOX and CAL *in vitro*. Consistent with our previous studies [21], DOX stimulation upregulated the expression of the pyroptotic executor GSDMD-N and cleaved-caspase-1. In addition, cells exposed to DOX showed significantly increased expression of NLRP3, ASC, IL-18, and IL-1*β*. CAL exerts its antipyroptosis effect in a dose-dependent manner, as it reduces DOX-induced expression of pyroptotic executor and pyroptotic-related proteins. These results suggest that CAL ameliorates DOX-induced cardiomyocyte pyroptosis in H9c2 cells.

Furthermore, C57BL/6J mice were used to examine the cardioprotective effects of CAL. Herein, we observed DOX-induced cardiotoxicity led to myocardial damage and decreased cardiac function, along with the overexpression of pyroptotic-related proteins, detectable by IHC and western blotting. These results suggest that cardiomyocyte pyroptosis occurs in DOX-treated mice. However, cotreatment of CAL with DOX alleviated myocardial injuries, improved cardiac function, and downregulated levels of pyroptosis-associated proteins in mice, suggesting that CAL afforded antipyroptotic effects. However, the molecular mechanism through which CAL inhibits cardiomyocyte pyroptosis remains unclear.

According to previous reports, inflammasomes can induce pyroptosis, among which NLRP3 inflammasome is the most widely explored, accompanied by caspase-1 activation [31]. Consistent with a previous report [13], our study found that DOX-induced cardiotoxicity was associated with the NLRP3 inflammasome. As previously reported, we observed the expression of the NLRP3 inflammasome in DOX-stimulated mice and H9c2 cells in the present study, manifested as the expression of NLRP3 inflammasome and pyroptosis-associated proteins. However, CAL decreased these proteins in a dose-dependent manner. Therefore, further studies are needed to confirm whether CAL inhibits cardiomyocyte pyroptosis during DOX-induced cardiotoxicity by regulating the NLRP3 inflammasome. To examine this hypothesis, we employed nigericin (a special stimulator of NLRP3) and NLRP3 overexpression. Interestingly, nigericin and NLRP3 overexpression reversed the inhibitory effect of CAL on pyroptosis. Meanwhile, nigericin and NLRP3 overexpression restored the expression of NLRP3-pyroptosis related molecules inhibited by CAL treatment. These results confirmed our hypothesis that CAL reduces cardiomyocyte pyroptosis by inhibiting the NLRP3 inflammasome.

## 5. Conclusion

In conclusion, the present confirmed that CAL alleviates DOX-induced cardiotoxicity and cardiomyocyte pyroptosis through the NLRP3-caspase-1-GSDMD pathway. However, this study has some limitations. First, some other inflammasomes, such as NLRP1b, NLRC4, AIM2, NLRP6, and NLRP9b [37], have also been reported to trigger pyroptosis, but the present study only focused on NLRP3-mediated pyroptosis. Second, the specific mechanism of CAL-mediated antioxidant activity and reduction of mitochondrial oxidative stress injury remains unclear. Therefore, we plan to explore the mechanism of pyroptosis in DOX-induced cardiotoxicity, as well as the mechanism of CAL-mediated antioxidant activity and reduction of mitochondrial oxidative stress.

## Data Availability

The data used to support the findings of this study are included within the article and the supplementary information file.

## Supplementary Materials

Supplementary Materials Supplementary Figure 1: CAL prevented DOX-induced oxidative stress and cardiomyocyte pyroptosis in H9c2 cells. Detection of ATP production (a), MDA content (b), SOD activity (c), and GSH-Px activity (d) in H9c2 cells (*n* = 6). (e)–(j) Quantitative analysis of NLRP3, ASC, caspase-1, IL-1*β*, IL-18, and GSDMD in H9c2 cells (*n* = 3). All data were represented as mean ± SEM. ^∗^*P* < 0.05, ^∗∗^*P* < 0.01, and ^∗∗∗^*P* < 0.001 compared with the control group, ^#^*P* < 0.05, ^##^*P* < 0.01, and ^###^*P* < 0.001 compared with the DOX group. Supplementary Figure 2: NLPR3 reversed the anticardiotoxicity effect of CAL *in vitro*. (a) Cytotoxicity of nigericin towards H9c2 cells. Cells were treated with 0-40 *μ*M nigericin for 24 h, and cell viability was detected by CCK-8 assay. (b) NLRP3 activation by nigericin enhanced the DOX-induced cytotoxicity. (c) NLRP3 activation by nigericin increased LDH release. (d) Changes of H9c2 cells morphology. (e) NLRP3 activation by nigericin increased IL-18 release. (f) NLRP3 activation by nigericin increased IL-1*β* release. (g) and (h) Immunofluorescence quantitative analysis showed nigericin increased NLRP3 and caspase-1 expression. All data were represented as mean ± SEM, *n* = 6. ^∗^*P* < 0.05 compared with the control group, ^#^*P* < 0.05 compared with the DOX group, ^@^*P* < 0.05 compared with the CAL + DOX group, ^&^*P* < 0.05 compared with the Nig + DOX group. Supplementary Figure 3: activation of NLRP3 with nigericin and overexpression of NLRP3 reversed the antipyroptotic effects of CAL *in vitro*. (a) Quantitative analysis of NLRP3, ASC, caspase-1, IL-1*β*, IL-18, and GSDMD in H9c2 cells. All data were represented as mean ± SEM, *n* = 3. ^∗^*P* < 0.05 compared with the control group, ^#^*P* < 0.05 compared with the DOX group, ^@^*P* < 0.05 compared with the CAL + DOX group, ^&^*P* < 0.05 compared with the Nig + DOX group. (b) Quantitative analysis of NLRP3, ASC, caspase-1, IL-1*β*, IL-18, and GSDMD in H9c2 cells. All data were represented as mean ± SEM, *n* = 3. ^∗^*P* < 0.05 compared with the control group, ^#^*P* < 0.05 compared with the DOX + pcDNA group, ^@^*P* < 0.05 compared with the CAL + DOX + pcDNA − NLRP3 group, ^&^*P* < 0.05 compared with the DOX + pcDNA − NLRP3 group. Supplementary Figure 4: CAL alleviated DOX-induced cardiac dysfunction and inflammation in mice. (a) HW/BW ratio in the different groups (*n* = 6). (b)–(e) CAL improved cardiac function in mice. Echocardiography of LVEF, LVFS, LVEDD, and LVESD in each group (*n* = 3). (f) CAL decreased serum BNP (*n* = 6). (g) CAL decreased serum LDH (*n* = 6). (h) CAL decreased CRP content (*n* = 6). (i) CAL decreased MCP-1 content (*n* = 6). Data were depicted as mean ± SEM. ^∗∗^*P* < 0.01, ^∗∗∗^*P* < 0.001 compared with the control group, ^#^*P* < 0.05, ^##^*P* < 0.01, and ^###^*P* < 0.001 compared with the DOX group.

## References

[1] Banke A., Fosbol E. L., Moller J. E. (2018). Long-term effect of epirubicin on incidence of heart failure in women with breast cancer: insight from a randomized clinical trial. *European Journal of Heart Failure*.

[2] Osataphan N., Phrommintikul A., Chattipakorn S. C., Chattipakorn N. (2020). Effects of doxorubicin-induced cardiotoxicity on cardiac mitochondrial dynamics and mitochondrial function: insights for future interventions. *Journal of Cellular and Molecular Medicine*.

[3] Wenningmann N., Knapp M., Ande A., Vaidya T. R., Ait-Oudhia S. (2019). Insights into doxorubicin-induced cardiotoxicity: molecular mechanisms, preventive strategies, and early monitoring. *Molecular Pharmacology*.

[4] Tadokoro T., Ikeda M., Ide T. (2020). Mitochondria-dependent ferroptosis plays a pivotal role in doxorubicin cardiotoxicity. *JCI Insight*.

[5] Kalyanaraman B. (2020). Teaching the basics of the mechanism of doxorubicin-induced cardiotoxicity: have we been barking up the wrong tree?. *Redox Biology*.

[6] Zeng C., Duan F., Hu J. (2020). NLRP3 inflammasome-mediated pyroptosis contributes to the pathogenesis of non- ischemic dilated cardiomyopathy. *Redox Biology*.

[7] Zhao L., Tao X., Qi Y., Xu L., Yin L., Peng J. (2018). Protective effect of dioscin against doxorubicin-induced cardiotoxicity via adjusting microRNA-140-5p-mediated myocardial oxidative stress. *Redox Biology*.

[8] Wu Y. Z., Zhang L., Wu Z. X., Shan T. T., Xiong C. (2019). Berberine ameliorates doxorubicin-induced cardiotoxicity via a SIRT1/p66Shc-mediated pathway. *Oxidative Medicine and Cellular Longevity*.

[9] Long G., Chen H., Wu M. (2020). Antianemia drug roxadustat (FG-4592) protects against doxorubicin-induced cardiotoxicity by targeting antiapoptotic and antioxidative pathways. *Frontiers in Pharmacology*.

[10] Lebrecht D., Geist A., Ketelsen U. P., Haberstroh J., Setzer B., Walker U. A. (2007). Dexrazoxane prevents doxorubicin-induced long-term cardiotoxicity and protects myocardial mitochondria from genetic and functional lesions in rats. *British Journal of Pharmacology*.

[11] Gu J., Huang H., Liu C. (2021). Pinocembrin inhibited cardiomyocyte pyroptosis against doxorubicin-induced cardiac dysfunction via regulating Nrf2/Sirt3 signaling pathway. *International Immunopharmacology*.

[12] Wang P., Wang M., Hu Y. (2021). Isorhapontigenin protects against doxorubicin-induced cardiotoxicity via increasing YAP1 expression. *Acta Pharmaceutica Sinica B*.

[13] Zhai J., Tao L., Zhang S. (2020). Calycosin ameliorates doxorubicin-induced cardiotoxicity by suppressing oxidative stress and inflammation via the sirtuin 1-NOD-like receptor protein 3 pathway. *Phytotherapy Research*.

[14] Yarmohammadi F., Hayes A. W., Karimi G. (2021). Natural compounds against cytotoxic drug-induced cardiotoxicity: a review on the involvement of PI3K/Akt signaling pathway. *Journal of Biochemical and Molecular Toxicology*.

[15] Lu X., Lu L., Gao L., Wang Y., Wang W. (2021). Calycosin attenuates doxorubicin-induced cardiotoxicity via autophagy regulation in zebrafish models. *Biomedicine & Pharmacotherapy*.

[16] Shi J., Gao W., Shao F. (2017). Pyroptosis: gasdermin-mediated programmed necrotic cell death. *Trends in Biochemical Sciences*.

[17] Kang R., Zeng L., Zhu S. (2018). Lipid peroxidation drives gasdermin D-mediated pyroptosis in lethal polymicrobial sepsis. *Cell Host & Microbe*.

[18] Tong Y., Wang Z., Cai L., Lin L., Liu J., Cheng J. (2020). NLRP3 inflammasome and its central role in the cardiovascular diseases. *Oxidative Medicine and Cellular Longevity*.

[19] Tavakoli Dargani Z., Singla R., Johnson T., Kukreja R., Singla D. K. (2018). Exosomes derived from embryonic stem cells inhibit doxorubicin and inflammation-induced pyroptosis in muscle cells. *Canadian Journal of Physiology and Pharmacology*.

[20] Meng L., Lin H., Zhang J. (2019). Doxorubicin induces cardiomyocyte pyroptosis via the TINCR-mediated posttranscriptional stabilization of NLR family pyrin domain containing 3. *Journal of Molecular and Cellular Cardiology*.

[21] Zhang L., Jiang Y. H., Fan C. (2021). MCC950 attenuates doxorubicin-induced myocardial injury in vivo and in vitro by inhibiting NLRP3-mediated pyroptosis. *Biomedicine & Pharmacotherapy*.

[22] Priya L. B., Baskaran R., Huang C. Y., Padma V. V. (2017). Neferine ameliorates cardiomyoblast apoptosis induced by doxorubicin: possible role in modulating NADPH oxidase/ROS-mediated NF*κ*B redox signaling cascade. *Scientific Reports*.

[23] Bock F. J., Tait S. W. G. (2020). Mitochondria as multifaceted regulators of cell death. *Nature Reviews. Molecular Cell Biology*.

[24] Wang Y., Gao W., Shi X. (2017). Chemotherapy drugs induce pyroptosis through caspase-3 cleavage of a gasdermin. *Nature*.

[25] Liang L., Tu Y., Lu J. (2019). Dkk1 exacerbates doxorubicin-induced cardiotoxicity by inhibiting the Wnt/beta-catenin signaling pathway. *Journal of Cell Science*.

[26] Prathumsap N., Shinlapawittayatorn K., Chattipakorn S. C., Chattipakorn N. (2020). Effects of doxorubicin on the heart: from molecular mechanisms to intervention strategies. *European Journal of Pharmacology*.

[27] Tavakoli Dargani Z., Singla D. K. (2019). Embryonic stem cell-derived exosomes inhibit doxorubicin-induced TLR4-NLRP3-mediated cell death-pyroptosis. *American Journal of Physiology. Heart and Circulatory Physiology*.

[28] Fang X., Wang H., Han D. (2019). Ferroptosis as a target for protection against cardiomyopathy. *Proceedings of the National Academy of Sciences of the United States of America*.

[29] Kaludercic N., Di Lisa F. (2020). Mitochondrial ROS formation in the pathogenesis of diabetic cardiomyopathy. *Frontiers in Cardiovascular Medicine*.

[30] Ait-Aissa K., Heisner J. S., Norwood Toro L. E. (2019). Telomerase deficiency predisposes to heart failure and ischemia-reperfusion injury. *Frontiers in Cardiovascular Medicine*.

[31] Wang Z. Q., Chen M. T., Zhang R., Zhang Y., Li W., Li Y. G. (2016). Docosahexaenoic acid attenuates doxorubicin-induced cytotoxicity and inflammation by suppressing NF-*κ*B/iNOS/NO signaling pathway activation in H9C2 cardiac cells. *Journal of Cardiovascular Pharmacology*.

[32] Tian Z., Li X., Ma Y. (2017). Quantitatively intrinsic biomimetic catalytic activity of nanocerias as radical scavengers and their ability against H2O2and doxorubicin-induced oxidative stress. *ACS Applied Materials & Interfaces*.

[33] Malik A., Kanneganti T. D. (2017). Inflammasome activation and assembly at a glance. *Journal of Cell Science*.

[34] Maayah Z. H., Takahara S., Dyck J. R. B. (2021). The beneficial effects of reducing NLRP3 inflammasome activation in the cardiotoxicity and the anti-cancer effects of doxorubicin. *Archives of Toxicology*.

[35] Whelan R. S., Kaplinskiy V., Kitsis R. N. (2010). Cell death in the pathogenesis of heart disease: mechanisms and significance. *Annual Review of Physiology*.

[36] Del Re D. P., Amgalan D., Linkermann A., Liu Q., Kitsis R. N. (2019). Fundamental mechanisms of regulated cell death and implications for heart disease. *Physiological Reviews*.

[37] Xue Y., Enosi Tuipulotu D., Tan W. H., Kay C., Man S. M. (2019). Emerging activators and regulators of inflammasomes and pyroptosis. *Trends in Immunology*.

