# Supplementary material for: Calycosin Alleviates Doxorubicin-Induced Cardiotoxicity and Pyroptosis by Inhibiting NLRP3 Inflammasome Activation
Source: Oxid Med Cell Longev. 2022 Jan 5;2022:1733834. doi: 10.1155/2022/1733834 (PMC8754606; doi:10.1155/2022/1733834)
Supplement: Supplementary Materials — Supplementary Figure 1: CAL prevented DOX-induced oxidative stress and cardiomyocyte pyroptosis in H9c2 cells. Detection of ATP production (a), MDA content (b), SOD activity (c), and GSH-Px activity (d) in H9c2 cells (n = 6). (e)–(j) Quantitative analysis of NLRP3, ASC, caspase-1, IL-1β, IL-18, and GSDMD in H9c2 cells (n = 3). All data were represented as mean ± SEM. ∗P < 0.05, ∗∗P < 0.01, and ∗∗∗P < 0.001 compared with the control group, #P < 0.05, ##P < 0.01, and ###P < 0.001 compared with the DOX group. Supplementary Figure 2: NLPR3 reversed the anticardiotoxicity effect of CAL in vitro. (a) Cytotoxicity of nigericin towards H9c2 cells. Cells were treated with 0-40 μM nigericin for 24 h, and cell viability was detected by CCK-8 assay. (b) NLRP3 activation by nigericin enhanced the DOX-induced cytotoxicity. (c) NLRP3 activation by nigericin increased LDH release. (d) Changes of H9c2 cells morphology. (e) NLRP3 activation by nigericin increased IL-18 release. (f) NLRP3 activation by nigericin increased IL-1β release. (g) and (h) Immunofluorescence quantitative analysis showed nigericin increased NLRP3 and caspase-1 expression. All data were represented as mean ± SEM, n = 6. ∗P < 0.05 compared with the control group, #P < 0.05 compared with the DOX group, @P < 0.05 compared with the CAL + DOX group, &P < 0.05 compared with the Nig + DOX group. Supplementary Figure 3: activation of NLRP3 with nigericin and overexpression of NLRP3 reversed the antipyroptotic effects of CAL in vitro. (a) Quantitative analysis of NLRP3, ASC, caspase-1, IL-1β, IL-18, and GSDMD in H9c2 cells. All data were represented as mean ± SEM, n = 3. ∗P < 0.05 compared with the control group, #P < 0.05 compared with the DOX group, @P < 0.05 compared with the CAL + DOX group, &P < 0.05 compared with the Nig + DOX group. (b) Quantitative analysis of NLRP3, ASC, caspase-1, IL-1β, IL-18, and GSDMD in H9c2 cells. All data were represented as mean ± SEM, n = 3. ∗P < 0.05 compared with the control group, # [file 1733834.f1.docx]

Supplementary Figure 1

Supplementary Figure 1: CAL prevented DOX-induced oxidative stress and cardiomyocyte pyroptosis in H9c2 cells. Detection of ATP production (a), MDA content (b), SOD activity (c), and GSH-Px activity (d) in H9c2 cells (n = 6). (e - j) Quantitative analysis of NLRP3, ASC, caspase-1, IL-1β, IL-18, and GSDMD in H9c2 cells (n = 3). All data were represented as mean ± SEM. ^*^*P* < 0.05, ^**^*P* < 0.01, ^***^ *P* < 0.001 compared with the control group, ^#^ *P* < 0.05, ^##^*P* < 0.01, ^###^*P* < 0.001 compared with the DOX group.

Supplementary Figure 2

Supplementary Figure 2: NLPR3 reversed the anti-cardiotoxicity effect of CAL *in vitro*. (a) Cytotoxicity of nigericin towards H9c2 cells. Cells were treated with 0-40 μM nigericin for 24 h, and cell viability was detected by CCK-8 assay. (b) NLRP3 activation by nigericin enhanced the DOX-induced cytotoxicity. (c) NLRP3 activation by nigericin increased LDH release. (d) Changes of H9c2 cells morphology. (e) NLRP3 activation by nigericin increased IL-18 release. (f) NLRP3 activation by nigericin increased IL-1β release. (g-h) Immunofluorescence quantitative analysis showed nigericin increased NLRP3 and caspase-1 expression. All data were represented as mean ± SEM, n = 6. ^*^*P* < 0.05 compared with the control group, ^#^ *P* < 0.05 compared with the DOX group, ^@^*P* < 0.05 compared with the CAL + DOX group, ^&^*P* < 0.05 compared with the Nig + DOX group.

Supplementary Figure 3

Supplementary Figure 3: Activation of NLRP3 with nigericin and overexpression of NLRP3 reversed the anti-pyroptotic effects of CAL *in vitro*. (a) Quantitative analysis of NLRP3, ASC, caspase-1, IL-1β, IL-18, and GSDMD in H9c2 cells. All data were represented as mean ± SEM, n = 3. ^*^*P* < 0.05 compared with the control group, ^#^ *P* < 0.05 compared with the DOX group, ^@^*P* < 0.05 compared with the CAL + DOX group, ^&^*P* < 0.05 compared with the Nig + DOX group. (b) Quantitative analysis of NLRP3, ASC, caspase-1, IL-1β, IL-18, and GSDMD in H9c2 cells. All data were represented as mean ± SEM, n = 3. ^*^*P* < 0.05 compared with the control group, ^#^ *P* < 0.05 compared with the DOX + pcDNA group, ^@^*P* < 0.05 compared with the CAL + DOX + pcDNA - NLRP3 group, ^&^*P* < 0.05 compared with the DOX + pcDNA - NLRP3 group.

Supplementary Figure 4

Supplementary Figure 4: CAL alleviated DOX-induced cardiac dysfunction and inflammation in mice. (a) HW/BW ratio in the different groups (n = 6). (b-e) CAL improved cardiac function in mice. Echocardiography of LVEF, LVFS, LVEDD, LVESD in each group (n=3). (f) CAL decreased serum BNP (n=6). (g) CAL decreased serum LDH (n = 6). (h) CAL decreased CRP content (n = 6). (i) CAL decreased MCP-1 content (n = 6). Data were depicted as mean ± SEM. ^**^*P* < 0.01, ^***^ *P* < 0.001 compared with the control group, ^#^ *P* < 0.05, ^##^*P* < 0.01, ^###^*P* < 0.001 compared with the DOX group.
